# Successful Treatment of Severe Post-craniotomy Meningitis Caused by an *Escherichia coli* Sequence Type 410 Strain Coharboring *bla*_*NDM*__–__5_ and *bla*_*CTX*__–__*M*__–__65_

**DOI:** 10.3389/fmicb.2021.729915

**Published:** 2021-09-08

**Authors:** Qing Yang, Piao-piao Zhang, Yan Jiang, Xiu-jue Zheng, Min Zheng, Ting-ting Qu

**Affiliations:** ^1^State Key Laboratory for Diagnosis and Treatment of Infectious Diseases, National Clinical Research Center for Infectious Diseases, Collaborative Innovation Center for Diagnosis and Treatment of Infectious Diseases, The First Affiliated Hospital, Zhejiang University School of Medicine, Hangzhou, China; ^2^Department of Laboratory Medicine, College of Medicine, The First Affiliated Hospital, Zhejiang University, Hangzhou, China; ^3^Department of Infectious Diseases, Sir Run Run Shaw Hospital, Zhejiang University School of Medicine, Hangzhou, China; ^4^Department of Neurological Surgery, College of Medicine, The First Affiliated Hospital, Zhejiang University, Hangzhou, China

**Keywords:** intracranial infection, *bla*_*CTX*__–__*M*__–__65_, treatment protocol, *bla*_*NDM*__–__5_, carbapenem-resistant *Escherichia coli*

## Abstract

Intracranial infections caused by multidrug-resistant Gram-negative bacterium have led to considerable mortality due to extremely limited treatment options. Herein, we firstly reported a clinical carbapenem-resistant *Escherichia coli* isolate coharboring *bla*_*NDM*__–__5_ and *bla*_*CTX*__–__*M*__–__65_ from a patient with post-craniotomy meningitis. The carbapenem-resistant *Escherichia coli* strain CNEC001 belonging to Sequence Type 410 was only susceptible to amikacin and tigecycline, both of which have poor penetration through the blood-brain barrier (BBB). The *bla*_*CTX*__–__*M*__–__65_ gene was expressed on a 135,794 bp IncY plasmid. The *bla*_*NDM*__–__5_ gene was located on a genomic island region of an IncX3-type plasmid pNDM5-CNEC001. Based on the characteristics of the strain, we presented the successful treatment protocol of intravenous (IV) tigecycline and amikacin combined with intrathecal (ITH) amikacin in this study. Intracranial infection caused by *Escherichia coli* coharboring *bla*_*NDM*__–__5_ and *bla*_*CTX*__–__*M*__–__65_ is rare and fatal. Continuous surveillance and infection control measures for such strain need critical attention in clinical settings.

## Introduction

Intracranial infection caused by carbapenem-resistant *Enterobacteriaceae* (CRE) is one of the most devastating complications following neurosurgery and also a serious nosocomial infection with high mortality (Fang et al., 2017). Limited antibiotics options due to the high resistance profile of CRE and poor blood-brain barrier (BBB) penetration complicate the treatment of CRE-related meningitis/encephalitis. The most common mechanism in CRE is carbapenemases production (Berger, 2011). New Delhi metallo-beta-lactamase (NDM) is a recently discovered metallo-beta-lactamase with enhanced carbapenem hydrolysis activity, especially NDM-5, and it can hydrolyze almost all beta-lactams except monobactams (Hornsey et al., 2011). NDM-5-producing *Escherichia coli* can lead to severe infections in diverse anatomical locations. Previous studies in China have shown several sporadic cases of clinical infections caused by *bla*_*NDM*__–__5_-positive *E. coli* including intestinal disease, urinary tract infections, invasive bloodstream and pulmonary infections (Zhu et al., 2016). Currently, intracranial infections due to *E. coli* producing NDM-5 and the related treatment experience are scarce. In this study, we report a case of secondary meningitis caused by an *E. coli* ST410 strain coproducing NDM-5 and CTX-M-65 for the first time, which was successfully treated by intravenous (IV) tigecycline and amikacin in combination with intrathecal (ITH) amikacin. Meanwhile, genomic and phenotypic characteristics of the strain are described in detail.

## Case Presentation

**FIGURE 1:**
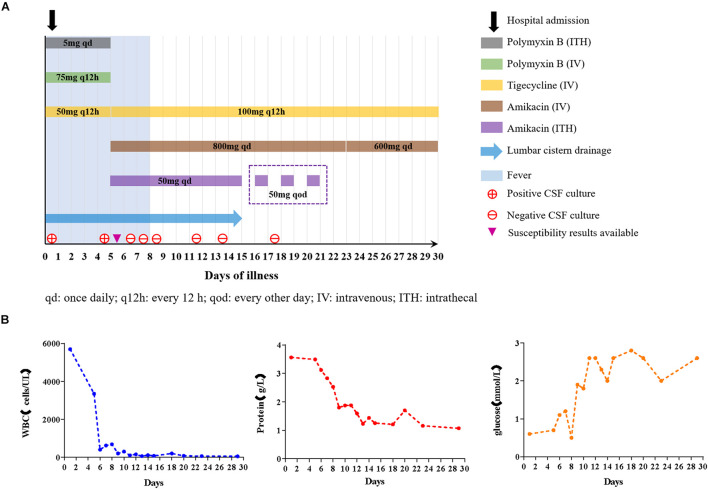
**(A)** Diagram of antibiotics administration, fever, and CSF cultures in the case during hospitalization. The contents in the box refer to the usage and dosage of each antibiotic at different periods. **(B)** Trends of CSF analytes after treatment.

A 67-year-old male, weighing 62 kg, was treated with intracranial hematoma clearance and bone flap decompression due to severe craniocerebral trauma caused by a traffic accident. The patient was transferred to the intensive care unit (ICU) after emergency surgery. The patient developed a fever of 38.1°C the day after surgery. Ten days later, consciousness disturbance was deteriorated; the culture of the cerebrospinal fluid (CSF) yielded carbapenem-resistant *E. coli*. Systematic tigecycline and intrathecal polymyxin B were used but did not improve CSF findings. The patient was transferred to our hospital for further treatment. The first CSF examination after admission revealed severe leukocytic pleocytosis (5,700 cells/μL, neutrophil accounted for 93%), elevated protein (2.82 g/L), and low glucose (0.7 mmol/L). Lumbar cistern drainage was performed immediately. The CSF exhibited pale yellow with turbidity. Antibiotics were changed to polymyxin B 75 mg × every 12 h (q12 h) IV + tigecycline 50 mg × every 12 h (q12 h) IV + polymyxin B 5 mg × once daily (qd) ITH. On day 6 after admission, we got the CSF bacterial culture and susceptibility results that *E. coli* was the pathogen, being resistant to many classes of antibiotics (including carbapenem) while only susceptible to tigecycline and amikacin. Due to severe neurotoxicity (newly emergent seizure) induced by polymyxin B, it was discontinued and was replaced with amikacin 800 mg × qd IV. Given that systemic anti-infection therapy alone may not achieve the effective concentration for antimicrobial activity in central nervous system (CNS), the patient was commenced on concurrent intrathecal administration of amikacin 50 mg × qd. As the poor activity of tigecycline to cross the BBB, we applied a higher dose of 100 mg × q12 h IV. From day 7, repeated CSF cultures were negative. The highest temperature during treatment was 39°C. From day 9, the patient had no fever. CSF cell count and protein content all followed a declining trend during the treatment process. A wide range of bacteria can produce biofilm on prosthetic implants and survive antimicrobial therapy. Hence, on day 15, the lumbar cistern drain was removed and intrathecal amikacin was adjusted to 50 mg × every other day (qod) ITH. The timeline of treatment options, disease course, and laboratory findings is shown in Figure 1. After further consolidation therapy, the patient recovered well and no recurrence was observed during the 1-year follow-up period.

## Materials and Methods

### Collection and Identification of Isolate

Strain CNEC001 was isolated from the patients’ cerebrospinal fluid sample and identified by an automated Vitek 2 compact system (bioMérieux, France).

### Antibiotic Susceptibility Testing

Susceptibility testings to tigecycline, ceftazidime/avibactam and polymyxin B were conducted by using the reference Clinical and Laboratory Standards Institute (CLSI) broth microdilution method (Clinical and Laboratory Standards Institute, 2020), while other antimicrobial agents were performed by Vitek 2 compact system and the Kirby--Bauer disk diffusion assay. Antibiotics tested in the study were ampicillin, piperacillin/tazobactam, ceftazidime, ceftriaxone, cefepime, ciprofloxacin, levofloxacin, fosfomycin, gentamicin, amikacin, aztreonam, imipenem, meropenem, ceftazidime/avibactam, tigecycline, and polymyxin B. The breakpoint of tigecycline was interpreted according to the criterion and recommendation from European Committee on Antimicrobial Susceptibility Testing^1^ while the remaining antimicrobial results were determined in accordance with Clinical and Laboratory Standards Institute (Clinical and Laboratory Standards Institute, 2020). *E. coli* ATCC 25922 was used as a control strain.

### Whole Genome Sequencing (WGS) and Bioinformatic Analysis

Whole DNA of the strain was extracted using a QIAamp DNA MiniKit (Qiagen, Valencia, CA, United States) according to the manufacturer’s protocol. Purified DNA samples were submitted to next-generation high-throughput sequencing (NGS) on the HiSeq2000^TM^ platform (Illumina, San Diego, United States) with 2^∗^100-bp paired-end reads. The overall genome coverage was 108 times and the N50 value of the contigs was 94.9 kbp, after assembling by the Illumina sequencing solely. The strain was further subjected to long-read high-throughput sequencing (LRS) on the MinION platform (Nanopore, Oxford, United Kingdom). Sequencing, libraries were prepared using the SQK-LSK109 Ligation Sequencing kit in conjunction with the PCR-Free ONT EXP-NBD104 and 114 Native Barcode Expansion kit, without optional shearing steps to select for long reads. Individual libraries were quantitated through Qubit. At last, the library was sequenced on a MinION platform. The overall genome coverage of the MinION sequencing was 176 times. We did not obtain the N50 value only for long read data because we used hybrid assembly method and the long reads were only utilized for closing the gap between the contigs that short reads assembled. We used Unicycler v0.4.8, an assembly pipeline, for our bacterial genome assembly with both short and long reads. Unicycler will first use SPAdes to make a short-read assembly graph, and then it will use various methods to scaffold that graph with the long reads. Several mapping strategies were used in Unicycler assembly pipeline for after-polishing to improve the assembly quality, including using the Racon to polish the assembly (Wick et al., 2017). Contigs were annotated by Prokka (version 1.14.5) combined with BLAST searches.^2^ Antibiotic resistance genes, acquired virulence genes, multi-locus sequence typing (MLST) and plasmid incompatibility groups were all detected using bioinformatics tools available on the CGE Server.^3^ Plasmid DNA sequences were collinearly analyzed using Easyfig 2.2.5 (Sullivan et al., 2011). Gene organization diagrams were drawn in the CGView Server (Larsen, 2013).

### Ethical Approval

This study has been reviewed and approved by the ethical research committee of the First Affiliated Hospital, College of Medicine, Zhejiang University. The ethics committee approved the waiver of the patient’s informed consent, with the justification that this was a retrospective study whose information was obtained from medical records and that the data were de-identified and anonymously analyzed.

## Results

### Antibiotic Susceptibility Results

Strain CNEC001 was identified as *E. coli* by the Vitek 2 automated system. Antimicrobial susceptibility test revealed that CNEC001 was resistant to the majority of antimicrobial agents, including ampicillin, piperacillin/tazobactam, ceftazidime, ceftriaxone, cefepime, ciprofloxacin, levofloxacin, fosfomycin, aztreonam, imipenem, meropenem, and ceftazidime/avibactam, intermediate to polymyxin B and gentamicin, while only susceptible to tigecycline and amikacin (Table 1).

**TABLE 1:** Antimicrobial susceptibility of the pathogen isolated from CSF of the patient.

Bacteria: *Escherichia coli*			
Antibiotics	MIC (μg/mL)	KB (mm)	Drug sensitivity
Ampicillin		6	R
Piperacillin/tazobactam	≥128		R
Ceftazidime	≥64		R
Ceftriaxone	≥64		R
Cefepime	≥32		R
Ciprofloxacin		6	R
Levofloxacin	≥8		R
Fosfomycin		6	R
Gentamicin		13	I
Amikacin	≤2		S
Aztreonam		6	R
Imipenem	≥16		R
Meropenem		13	R
Ceftazidime/avibactam	≥256		R
Tigecycline	≤0.5		S
Polymyxin B	2		I

### Genomic Characteristics of CNEC001

Strain CNEC001 belonged to sequence type 410 (ST410). The genome size of CNEC001 was 5,034,429 bp, with 50.6% GC content. This strain carried four different plasmids and harbored multiple resistance genes, including three β-lactam resistance genes (*bla*_*NDM*__–__5,_ *bla*_*CTX*__–__*M*__–__65,_ *bla*_*CMY*__–__2_), two aminoglycoside resistance genes [*aph (4)- Ia*, *aadA5*], three sulphonamide resistance genes (*sul1*, *sul2*, *dfrA17*), one macrolide resistance gene [*mph(A)*] and one tetracycline resistance gene [*tet (B)*]. Most of these genes [*bla*_*CTX*__–__*M*__–__65_, *sul1*, *sul2*, *dfrA17, aph (4)- Ia, aadA5*, and *mph(A)*] were found on a 135,794 bp IncY plasmid. *bla*_*NDM*__–__5_ was located on a 46,161 bp IncX3 plasmid, which here was assigned pNDM5-CNEC001. *bla*_*CMY*__–__2_ was the only resistance gene mediated by chromosome. Virulence gene *terC* (Tellurium ion resistance protein) may be correlated with significant pathogenic potential of *E. coli*.

### Characteristics of the *Bla*_*NDM*__–__5_-Harboring Plasmid pNDM5-CNEC001

**FIGURE 2:**
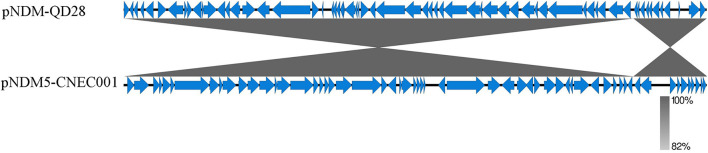
Comparison of the genetic organization between pNDM5-CNEC001 and pNDM-QD28 (GenBank accession number KU167608.1). Genes are denoted by arrows. Shading indicates regions of homology.

**FIGURE 3:**
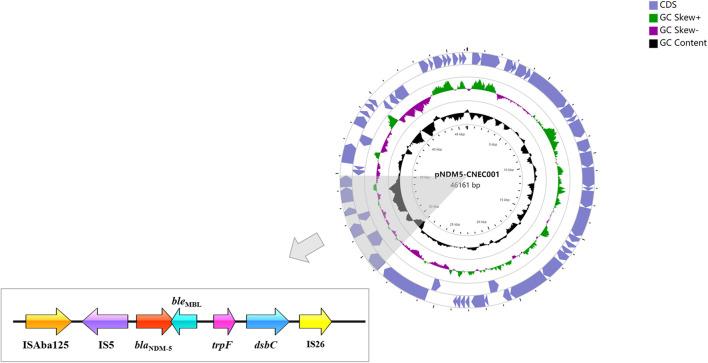
The genetic surroundings of *bla*_*NDM*__–__5_ in the plasmid pNDM5-CNEC001.

Plasmid pNDM5-CNEC001 was 46161 bp in size with average G+C content of 46.7%, encoding 63 predicted open reading frames (ORFs). It belonged to the IncX3 incompatibility group based on PlasmidFinder analysis and carried the sole resistance gene *bla*_*NDM*__–__5_. Sequence alignments on BLAST revealed that pNDM5-CNEC001 was highly similar to pNDM-QD28 (GenBank accession number KU167608.1), a plasmid carried by an NDM-5-producing *E. coli* ST167 in China, with more than 99.99% identities and 100% query coverage (Figure 2). Gene *bla*_*NDM*__–__5_ was within the typical structure IS*Aba125*-IS*5*-*bla*_*NDM*__–__5_-*ble*_*MBL*_-*trpF*-*dsbC*-IS*26* (Figure 3). pNDM5-CNEC001-like plasmids could be found in various *Enterobacteriaceae* isolates among which *E. coli* appeared to be particularly common, followed by *Klebsiella pneumoniae*.

### Nucleotide Sequence Accession Number

The complete genome sequence of strain CNEC001 has been deposited at DDBJ/ENA/GenBank under the accession JAIELB000000000. The version described in this paper is version JAIELB010000000. The complete nucleotide sequence of plasmid pNDM5-CNEC001 was submitted to GenBank with the accession number MZ270636.

## Discussion

To date, *E. coli* ST167 is classified as the predominant clonal lineage spreading the *bla*_*NDM*__–__5_ gene in China (Bi et al., 2018; Xu et al., 2019; Li et al., 2020). *E. coli* ST410 has been increasingly reported as NDM-5 or OXA-181-type carbapenemase producer, this lineage has been categorized as an internationally emerging “high-risk” clone evidenced by its expression of various antimicrobial resistance determinants (ESBLs, pAmpCs, carbapenemases gene, colistin resistance genes), effective interspecies transmission, persistence in hosts and enhanced pathogenicity (Roer et al., 2018). Humans, food animals, and the environments are all important sources of ST410 *E. coli* (Feng et al., 2019). Comparison between NDM-5-producing ST167 and ST410 *E. coli* isolates previously reported in China reveals that NDM-5-producing ST167 *E. coli* strains were recovered from more clinical samples (including ascites, sputum, urine, blood, pus, CSF, bile, rectal swab). NDM-5-producing ST410 *E. coli* have rarely reported in CSF (Hammerum et al., 2015; Krishnaraju et al., 2015; Wailan et al., 2015; Zhu et al., 2016; Li et al., 2018). Moreover, ST410 *E. coli* were more common to carry *bla*_*CMY*_.

In this study, according to the guideline established by Clinical and Laboratory Standards Institute (2020) which eliminated the “susceptible” interpretive category for the polymyxins, the ST410 *E. coli* strain CNEC001 was intermediate to polymyxin B and gentamicin, only susceptible to tigecycline and amikacin, and possessed virulence gene*terC* that enabled it to cause disease in the host. The tellurite resistance gene is widely spread among pathogenic species. Ter proteins play an important role in phagocytosis inhibition, allowing infective pathogens to evade the neutrophil responses (Bai et al., 2012). Apart from carbapenem resistance, CNEC001 showed aztreonam and cephalosporins resistance, which could be attributed to *bla*_*NDM*__–__5_ and *bla*_*CTX*__–__*M*__–__65_. Carbapenemase-encoding gene *bla*_*NDM*__–__5_ of strain CNEC001was detected on an IncX3-type plasmid pNDM5-CNEC001. IncX3-type plasmid is self-transmissible and has been proved as a common vehicle facilitating the horizontal transmission of either *bla*_*NDM*__–__5_ or *bla*_*OXA*__–__181_ between diverse species and hosts (Yang et al., 2014). BLAST analysis revealed that the IncX3 plasmid carrying *bla*_*NDM*__–__5_ in our study was highly similar to each other isolated from different countries and host sources, suggesting its ability to be an efficient vehicle for *bla*_*NDM*__–__5_ dissemination among humans, animals, foods and the environments, potentially indicating its role in the rapid spread of *bla*_*NDM*__–__5_-harboring isolates. The *bla*_*CTX*__–__*M*__–__65_ gene is also encoded on an easily transferable plasmid carrying multiple resistance genes of other classes of antibiotics, which is consistent with the results of previous studies (Bevan et al., 2017; Karami et al., 2021), leading to multidrug resistance if the plasmid is horizontally spread. CTX-M beta-lactamases are the most common types of Extended spectrum beta-lactamases (ESBLs), which can inactivate penicillins, cephalosporins, and aztreonam. The *bla*_*NDM*_ genes generally coexist with *bla*_*CTX*__–__*M*_ genes (Suay-Garcia and Perez-Gracia, 2019; Tooke et al., 2019). A study of the epidemiology of carbapenem-resistant *E. coli* in Chinese patients showed that almost all (>96%) NDM-producing strains encoded CTX-M-type β-lactamases (Tian et al., 2020). Once this occurs, it will lead to increased difficulty of antibiotic treatment, aztreonam being ineffective. There are few relevant reports on *E. coli* harboring the *bla*_*CTX*__–__*M*__–__65_ gene isolated from human in China. *bla*_*CTX*__–__*M*__–__65_ gene is widely found in *E. coli* isolated from food-producing animals and no sequence type related to the widespread popularity of CTX-M-65-type ESBL has been found. Previous study showed that *E. coli* ST410 encoding *bla*_*CTX*__–__*M*__–__65_ had been obtained from healthy broiler chickens (Liu et al., 2020). It is very likely that poultry will be sources of *bla*_*CTX*__–__*M*__–__65_ and transfer this resistant gene to human via food supply chain. We previously reported a community-acquired renal abscess caused by a ST410 *E. coli* strain coharboring *bla*_*NDM*__–__5_ and *bla*_*CTX*__–__*M*__–__65_ in an outpatient (Hu et al., 2020). However, to our knowledge, this is the first report of a clinical *E. coli* ST410 co-harboring *bla*_*NDM*__–__5_, *bla*_*CTX*__–__*M*__–__65_ and *bla*_*CMY*__–__2_ isolated from CSF. In this study, our patient had a history of head trauma that was treated with craniotomy and developed meningitis soon after emergent surgery, thus, we speculate that strain CNEC001 may originate from the surgical site colonized by the pathogenic bacteria which translocated into CNS during surgery and thus caused the infection. However, community-acquired resistant isolates might become epidemic in healthcare settings and cause hospital outbreaks, presenting a serious threat to inpatients’ health. Increased surveillance is still urgently required to prevent the emergence and further dissemination of the “superbug” which has co-production of ESBLs and NDM.

The optimal antimicrobial treatment regimen at present for MDR bacterial meningitis still stands unclear but should be based on *in vitro* drug sensitivity testing. Currently, drugs with inability to adequately permeate through the BBB (including amikacin, polymyxin B) are clinically used for intrathecal therapy of CNS infections caused by MDR Gram-negative pathogens (Tunkel et al., 2017; Molinaro et al., 2018; Nau et al., 2020). Intrathecal/intraventricular polymyxins combined with systemic administration were recommended to treat intracranial infection caused by CRE in recent several studies (Chen et al., 2020; Zhong et al., 2020). However, nephrotoxicity and neurotoxicity remain major problems for the clinical use of polymyxins. Intrathecal/intraventricular polymyxins therapy has been associated with serious neurological adverse effects including seizures, chemical meningitis and cauda equina syndrome. In order to improve clinical outcomes, polymyxins should be chosen in combination with other active antimicrobials whenever possible in treatment of CNS infections (Paul et al., 2018; van Duin et al., 2018; Pogue et al., 2020). When alternative drugs are available, it is strongly recommended to prioritize non–polymyxin drugs with antibacterial activity against CRE *in vitro*, including ceftazidime/avibactam (ineffective against bacteria producing metallo-beta-lactamase), dual carbapenems, aminoglycosides and tigecycline. Aminoglycosides gentamicin and amikacin are effective against approximately 50% of CRE isolates (Satlin et al., 2011) and still considered first-line therapy for CRE (Suay-Garcia and Perez-Gracia, 2019), however, they have disadvantages of nephrotoxicity and low penetration of CSF.

In our study, the pathogen was intermediate to gentamicin and polymyxin B and only susceptible to amikacin and tigecycline. The patient developed new adverse reaction (epilepsy) caused by polymyxin B during therapy, so treatment regimen we performed was i.v. tigecycline and i.v. amikacin plus intrathecal amikacin. Tigecycline is characterized as a time-dependent agent with concentration-dependent killing and drug-induced prolonged effects. The recommended intravenous regimen of tigecycline is an initial dose of 100 mg, followed by 50 mg every 12 h. Despite its high effectiveness against MDR pathogens, tigecycline is currently not recommended in cases of intracranial infection, as CSF concentrations ranged from only 0.035 to 0.048 mg/L with the usual intravenous dose of 100 mg/day in the absence of meningeal inflammation (Nau et al., 2020). We used a higher dose of 100 mg IV q12 h in order to obtain tigecycline concentration in excess of the MIC (0.5 μg/ml) for *E. coli* in CSF. Unfortunately, we did not monitor therapeutic drug concentrations while on therapy. Several cases previously reported failed to achieve desired target site concentrations (those exceeding the MIC) in CNS by utilizing double the recommended dosage (i.e.,100 mg IV Q12 h) (Chen et al., 2007; Ray et al., 2010). Given the strain was also susceptible to amikacin, hence, intravenous combined with intrathecal administration of amikacin were added to improve therapeutic effect. Amikacin is concentration-dependent antibiotic with prolonged post-antibiotic effect. CSF concentrations with uninflamed meninges are close to the MICs of moderately susceptible bacteria under usual intravenous dose (600 mg qd) (Nau et al., 2018). In our case, a higher intravenous dose of amikacin (800 mg × qd) and intrathecal administration (50 mg × qd) were performed in the initial stages of treatment to improve the effective concentration for antimicrobial activity in CNS. A systematic review covering the years 1946–2015 reported intrathecal doses ranging from 5 to 50-mg amikacin daily to be effective and well tolerated, though the optimum dosage still remains unclear (Nau et al., 2018).

Monotherapy with intrathecal drugs rarely induces durable responses. Intravenous combined with intrathecal administration can provide a high local drug concentration at the site of infection and has been demonstrated to be effective by recent research with successful outcomes (De Bonis et al., 2016; Nau et al., 2018). Combined antibiotic therapy can increase antimicrobial activity and yield better results than monotherapy. Early indwelling drainage of CSF is beneficial in alleviating intracranial infection, however, it is crucial for successful treatment that removing the prostheses as soon as possible after the CSF cultures have been negative as well as CSF and clinical parameters have improved.

## Conclusion

In conclusion, this is the first report of a clinical *E. coli* ST410 co-harboring *bla*_*NDM*__–__5_, *bla*_*CTX*__–__*M*__–__65_, and *bla*_*CMY*__–__2_ responsible for intracranial infection in China, which renders therapeutic intervention quite difficult and leads to poor outcome. This study also provides treatment experience that may apply to patients under similar circumstances, and unravels possible mechanisms of dissemination of antibiotic resistance genes. Constant and careful surveillance for NDM and ESBL-producing strains is urgently warranted in clinical settings. Prompt and appropriate infection control measures should be taken after the first isolation of such species.

## Data Availability Statement

The data presented in the study are deposited in the GenBank repository, accession numbers JAIELB000000000 and MZ270636. Our data is publicly available.

## Author Contributions

QY and P-pZ conceived the idea and performed the experiments. QY, P-pZ, and X-jZ analyzed the data. YJ helped with materials and reagents. QY wrote the manuscript. T-tQ and MZ reviewed the manuscript. All authors have read and agreed to the published version of the manuscript.

## Conflict of Interest

The authors declare that the research was conducted in the absence of any commercial or financial relationships that could be construed as a potential conflict of interest.

## Publisher’s Note

All claims expressed in this article are solely those of the authors and do not necessarily represent those of their affiliated organizations, or those of the publisher, the editors and the reviewers. Any product that may be evaluated in this article, or claim that may be made by its manufacturer, is not guaranteed or endorsed by the publisher.

## Footnotes

1
http://www.eucast.org/clinical_breakpoints

2
www.ncbi.nlm.nih.gov/blast/

3
https://cge.cbs.dtu.dk/services/
